# The impact of amplification on quality of life in women with Turner syndrome

**DOI:** 10.1186/s13023-024-03122-z

**Published:** 2024-03-13

**Authors:** Lauren Mann, Lindsey VanLooy

**Affiliations:** 1 University of Kansas Medical Center, 3901 Rainbow Blvd MS 3047, 66160 Kansas City, KS USA; 2 University of North Carolina Hospitals, Chapel Hill, NC USA

**Keywords:** Turner syndrome, Aural rehabilitation, Hearing loss, Hearing aids, Quality of life

## Abstract

**Background:**

Individuals with Turner syndrome (TS, ORPHA 881) experience barriers in communication throughout life as they navigate both early conductive, and progressive sensorineural hearing loss amid other healthcare needs. Hearing loss is self-identified as one of the largest unmet healthcare needs.

**Purpose:**

The purpose of this study was to investigate the impact of treatment for hearing loss on communication confidence and quality of life measures for individuals with TS.

**Research design:**

We employed a prospective cross-sectional study design that included both online survey data and audiometric data for a subset of participants.

**Study sample:**

We recruited 179 adults with TS at the Turner Syndrome Society of the United States (TSSUS) Conference, and through a variety of regional TS organizations’ social media platforms. Audiological data was collected onsite at the conference for a subset of 67 participants; 8 of which who were followed after receiving subsequent treatment with hearing aids.

**Data collection and analysis:**

The online survey design included demographic questions, the Communication Confidence Profile (CCP), and the RAND 36-Item Health Survey 1.0. Audiometric data included tympanometry, puretone air, and puretone bone conduction thresholds. Descriptive statistics, parametric, and non-parametric tests were used to analyze both survey and audiometric data.

**Results:**

74% of participants had a self-reported diagnosis of hearing loss, of which 61% were previously recommended amplification. Only 38% of participants reported using hearing aids. For those participants who wore hearing aids, Total CCP Score, ‘Confidence in Ability to Hear Under Various Conditions’, and ‘Energy/Vitality’ metrics were significantly greater than those with untreated hearing loss warranting a hearing aid. Collectively, Total CCP Score and ‘Confidence in Ability to Hear Under Various Conditions’ increased significantly when participants were fit with hearing aids.

**Conclusion:**

The results support previous data where hearing loss is a self-identified healthcare concern among women with Turner syndrome, yet many fail to receive appropriate hearing evaluation or treatment. Additionally, the use of hearing aids may improve communication confidence and quality of life in women with Turner syndrome. Furthermore, this study confirms the need for long-term audiological care and monitoring in women with Turner syndrome.

## Background

Turner syndrome (TS, ORPHA 881) is a sex chromosome abnormality (SCA) that occurs in approximately 1 in 2000–2500 live female births because of a partial or total loss of the X-chromosome on some or all the body’s cells [1]. Resulting clinical presentation of TS is dependent on the affected region of the X-chromosome. The most common karyotype affecting roughly 50% of persons postnatally involves monosomy (45,X). Alternately, cases present as mosaic karyotype 45,X/46,XX or present structurally abnormal X-chromosomes or duplication. This includes 46,X,i(Xq), 46,XX, 47,XXX, 46,X,del(Xp), or 46,XY [2, 3]. Loss of Xp or Xq is often expressed as TS but duplications are often phenotypically normal with less than 10% presenting with a duplication (isochromosome) of the long arm of one X (46,X,i(Xq)) [4].

With the primary karyotype, X-chromosome loss occurs on all cells and presentation includes heart defects, infertility, short-stature, differences in craniofacial development and a variety of otologic disorders among other possible characteristics [5]. Reduced height and lymphoedema occur at high rates. Reduced height and skeletal abnormalities are associated with the short stature homeobox-containing gene (SHOX) [1, 2]. Lymphoedema of the hands, feet and neck associated with the putative lymphogenic gene is shown in around 60% of persons [6].

Individuals with mosaic karyotype may present with both a 46,XX population that can reduce the severity of a monosomic phenotype. Hence, mosaic TS generally includes similar, but less severe health concerns than classic monosomy presentation. Other cytogenetic abnormalities including a range of inversions, rings, deletions, duplications, and translocations are associated with various phenotypes [4]. Still, individuals in both groups are at a considerable risk of developing both transient middle ear infections and progressive, permanent, sensorineural hearing loss [7]. For this reason, the standard of care for individuals with TS includes full audiological evaluations every three to five years [5]. Not only is this the standard of care, individuals with TS also self-identify a significant need for audiological management. Data from a 2016 survey (*n* = 1386) showed hearing loss was the most reported physical health condition impacting everyday life in adults [5].

In the general adult population with up to a moderate severity of hearing loss, hearing aids have been shown to significantly improve hearing specific quality of life. These factors include the ability to take part in everyday situations, as well as ability to effectively listen to others. Importantly, those with hearing loss treated with hearing aids show a significant increase in general health-related quality of life measures compared to unaided controls [8]. Given the lifespan of hearing-related issues in the TS population, duration of hearing aid use is an important point to consider when evaluating the impact of hearing aid use on quality of life. In a sample of 51 patients in California with untreated hearing loss, a statistically significant improvement in communication confidence scores was measured just 2 to 4 weeks following hearing aid fitting [9]. This suggests that the interventional effects of hearing aid use are seen rapidly, which is important when considering a population like those with TS, who are often facing complex and numerous health care needs.

Early identification and intervention of hearing loss may offer individuals with TS improved quality of life and improved communication with their healthcare team. In general, untreated hearing loss contributes to lower adherence to medical recommendations, higher healthcare costs, and higher risk of hospital readmission compared to patients without hearing loss [10].

At present, there are no known studies that have assessed the effects of hearing aid use on quality of life, specifically in the Turner syndrome population. Given the higher rates of psychological comorbidities seen in this population, it is unclear whether hearing aids alone will be sufficient for improving quality of life and communication confidence. There is an overall lack of subjective data related to the impact of hearing loss on quality of life in individuals with Turner syndrome, despite hearing loss being a self-defined priority.

The purpose of this study was to investigate communication confidence and quality of life outcomes for individuals with Turner syndrome that have hearing loss and are treated with hearing aids versus those that are hearing aid candidates but untreated. Given that much of the research on hearing loss in Turner syndrome has been completed in Sweden and the United Kingdom, this study additionally sought to collect information on the usage of audiological services and hearing aids in adults with TS living in the United States.

## Methods

This research was approved by the University of Kansas Medical Center (KUMC) Human Research Protection Program; Study: 00144161. Study funding was provided by Turner Syndrome Global Alliance and Global Genes. The authors have no additional financial interests to disclose.

### Participants

Participants (*n* = 179) were recruited from the Turner Syndrome Society of the United States (TSSUS) annual conference in Nashville Tennessee (*n* = 67) and via online social media groups (*n* = 112). All participants completed a 10-15-minute REDCap questionnaire that included: demographic questions, The Communication Confidence Profile (CCP), and the RAND 36-Item SF Health Survey (RAND SF-36). Participants recruited in-person at the annual TSSUS conference, underwent puretone threshold testing in addition to completing the online survey. Conference participants were excluded in puretone data collection if they were under the age of 18, had occluding cerumen in one or both ears, or declined to consent to participation. Presence of cerumen was not an exclusion criterion for participation in the survey responses given the hybrid in-person and online recruiting efforts. All conference attendees were offered a hearing evaluation at no cost regardless of participation in this research.

Study participants all indicated they were female with a mean age of 39.73 years (range 18 to 68 years). Participants were informed of their hearing status and whether hearing aids were advised by a licensed audiologist on the study team. No participants directly received amplification in this study, but they were all provided with information about The Hearing Aid Project (HAP), a national non-profit organization that provides refurbished hearing aids at no cost to individuals in need. As part of this research, the HAP assisted all interested participants in applying for hearing aids and connecting them with a qualified audiologist in their area.

### Survey protocol

All participants completed the study surveys at least once, with a subset of participants asked to complete the protocol a second time following hearing loss intervention.

### Demographic questions

Participants were asked to provide personal information including age, educational status, employment status, subjective hearing difficulties, and otologic medical history.

### Communication confidence profile (CCP)

To gather specific information regarding participant confidence in a range of auditory communication skills, the CCP was utilized. This questionnaire includes 12 items arranged on a Likert-scale from ‘not at all’ [1] to ‘extremely’ [5] with possible total scores ranging from 12 to 60. Higher total CCP scores indicate more confidence in communication ability overall [9]. This survey evaluates two main constructs: (1) general confidence in the ability to hear under different listening conditions and (2) confidence in being able to improve hearing skills using devices or strategies. The CCP has been shown to have good reliability for both participants with and without hearing loss [9]. The CCP as a subjective measure of communication confidence, is moderately correlated with objective audiological measurements like pure tone average and word recognition scores [9]. This questionnaire provides 3 final scores: total CCP score, confidence in ability to hear in various environments, and utilization of communication strategies and devices (See: Table 1).

**Table 1:** Communication confidence profile clinical interpretation RAND 36-Item SF health survey (Version 1.0)

Total CCP Score	Clinical Interpretation
50+	Confident
40–50	Cautiously Certain
30–39	Tentative
29 and below	Insecure

At present, a Turner syndrome-specific quality of life (QoL) survey does not exist. However, according to a large systematic review of QoL measurements in the population with Turner syndrome, the RAND SF-36 was the most common and considered the most appropriate QoL measurement in current use [11]. The RAND SF-36 was developed by the RAND corporation using data from the Medical Outcomes Study [12]. This 36-item questionnaire contains 8 scales with a higher score indicating better health. Since the current study emphasized the psychosocial aspects of health, the following 5 scales were assessed: Social Functioning, Emotional Well-Being, Energy, General Health, and Role Limitations Due to Emotional Problems [12]. Scoring of the RAND 36-Item SF Health Survey followed the standard procedure of re-coding items, scoring them from 0 to 100, higher score being a more favorable health state, and averaging items to form the scaled scores. By nature of the online REDCap survey, no items could be left blank.

### Audiometric evaluation

Prior to audiometric testing, otoscopy was performed and conference participants with occluding cerumen were not included in this data (*n* = 6). Puretone air and bone audiometric thresholds were recorded using the Path Medical Sentiero portable audiometer. The audiological equipment was provided by The University of Kansas Medical Center Department of Hearing & Speech and was current regarding annual ANSI calibration standards. Puretone thresholds were measured using the Modified Hughson-Westlake procedure at standard octaves (250–8000 Hz) under supra-aural headphones and using a bone-conduction headband in a quiet room free of distractions. Regular measures of ambient noise were taken using a NIOSH Sound Level Meter to ensure the room didn’t exceed 40 dBA.

**Fig. 1:**
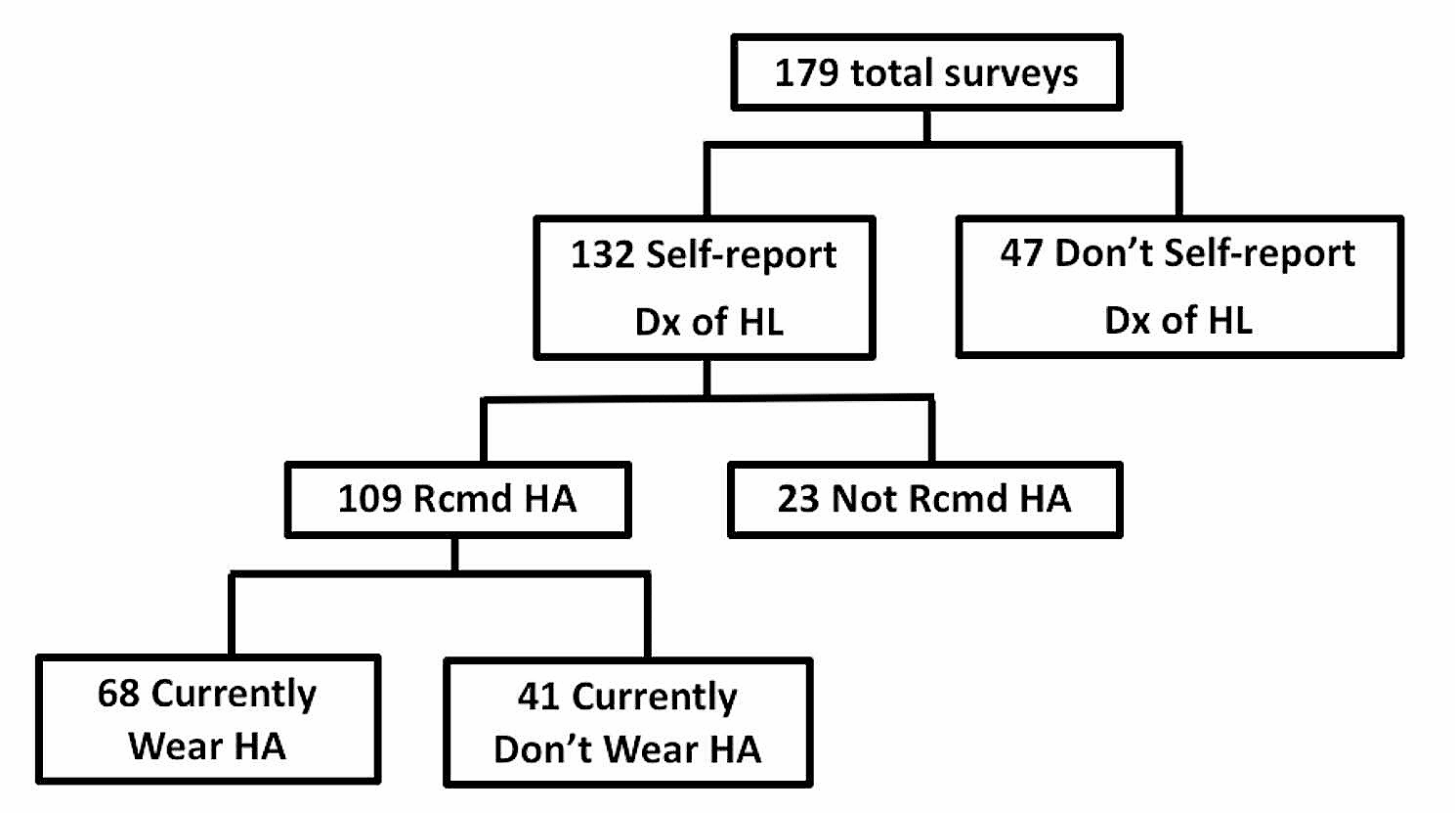
Hearing aid use

Normal hearing was quantified as thresholds at or lower than 20 dB HL at each test frequency 250 to 8000 Hz. A conductive component was defined by air-bone gaps of 15 dB HL or greater at any frequency. Puretone averages were calculated as the average of the air conduction thresholds at 500, 1000, and 2000 Hz. An aidable hearing loss was quantified as thresholds of 40 dB HL or greater at 500, 1000, 2000, 3000, or 4000 Hz; or hearing thresholds 26 dB HL or greater at 3 of these frequencies per the Department of Veterans Affairs 2012 guideline for determining disability due to hearing impairment [13]. A summary of self-reported hearing loss and ultimate hearing aid use is shown in Fig. 1.

### Statistical analysis

Statistical analysis was conducted using IBM SPSS Statistics for Windows [14]. Prior to full data analysis, missing survey data (8.9%) was handled by mean imputation. Audiological characteristics and hearing loss progression were evaluated using descriptive statistics. Differences in CCP and the RAND SF-36 scores between hearing aid (HA) users and non-HA users were assessed using independent sample t-tests or Mann Whitney U tests [15, 16]. This was dependent upon whether the samples were normally distributed, relative to the Shapiro-Wilk test. Change in CCP and RAND SF-36 scores for those fit with HAs was assessed using non-parametric Wilcoxon Signed Ranks test given the small sample size. All significance tests were two-tailed and conducted at the 5% significance level. No Type I error adjustment was made for multiple testing since all tests were considered explorative in nature. Effect sizes were calculated for all measurements. Cohen’s d values of 0.2 were considered a small effect, 0.5 medium, and 0.8 large. An r value of 0.1 was considered a small effect, 0.3 was considered medium, and 0.5 or greater was considered a large effect.

## Results

Demographic responses are outlined in Table 2 and included participants from 28 US states. For survey data, 74% (*n* = 132) of participants self-reported a previously diagnosed hearing loss overall and 61% (*n* = 109) reported a diagnosed hearing loss that warranted a hearing aid recommendation. Approximately half of the participants reported a history of at least one ear surgery. For those with hearing loss, 83% reported bilateral while 17% reported unilateral hearing loss. Like previous data, we found high rates of ear infections with 51% having greater than 5 ear infections in their lifetime. Only 38% of respondents reported current hearing aid use despite 61% reporting a diagnosed hearing loss that warranted a hearing aid recommendation. When asked about hearing aid use, 64% reported thinking hearing aid use would improve or greatly improve their quality of life. The 3 factors identified as most negatively impacted by hearing loss were: work ability overall, telephone use, and ability to talk in groups.

**Table 2:** Participant demographics

	Count	Percent
Occupation		
Unemployed	15	8.4%
Part-Time Student	2	1.1%
Full-Time Student	13	7.3%
Part-Time Employee	20	11.2%
Full-Time Employee	105	59.2%
Part-Time Employee/Student	5	2.8%
Retired	8	4.5%
Disabled	10	5.6%
Education		
High School/GED	48	26.8%
Associate’s Degree	26	14.5%
Bachelor’s Degree	65	36.3%
Graduate Degree	40	22.3%
Self-Report Diagnosis of Hearing Loss		
No	47	26.3%
Yes	132	73.7%
Ear With Hearing Loss		
Left	10	7.5%
Right	13	9.7%
Both	111	82.8%
History of Ear Surgery		
No	89	49.7%
Yes	90	50.3%
Number of Past Ear Infections		
None	14	7.8%
1 to 5	25	14.0%
5 to 10	21	11.7%
10 to 20	21	11.7%
More than 20	49	27.4%
Unsure	49	27.4%
Currently Wears Hearing Aids		
No	111	62.0%
Yes	68	38.0%
Age of Hearing Ad Device(s)		
1 year or newer	20	11.2%
2–5 years old	45	25.1%
Over 5 years old	3	1.7%

For participants with a diagnosed hearing loss that warranted a hearing aid recommendation (*n* = 109), those currently wearing hearing aids scored significantly higher on Total CCP (*p* = 0.039, d = 0.42) and on the ‘Confidence in Ability to Hear Under Various Conditions’ subscale (*p* = 0.03, d = 0.43) compared to those with untreated hearing loss. Hearing aid users also scored significantly higher on the ‘Energy/Vitality’ subscale (*p* = 0.022, d = 0.46) of the RAND SF-36. Age was significantly higher (*p* = 0.006) for current HA-users than non-users; with a mean of 46 and 40 years respectively.

On average, all participants, regardless of HA use, fell in the “tentative” category of communication confidence, indicating both groups might still benefit from intervention (See Table 3).

**Table 3:** Diagnosed hearing loss warranting a hearing aid recommendation

Measure	n	Mean(SD)	Median	t	Z	Effect Size	*P*-Value
Total CCP Score HA No HA	68 41	35.43(6.45) 32.83(6.02)	36 32	2.09		d = 0.42	0.039*
Confidence in Ability to Hear Under Var. Cond. HA No HA	68 41	28.08(6.42) 25.24(6.64)	29 25	2.21		d = 0.43	0.030*
Confidence in Ability to Improve Hearing Skills Using Strategies/Devices Hearing Aid No Hearing Aids	68 41	7.36(1.47) 7.57(1.68)	8 7		0.391	*r* = 0.04	0.696
Social Functioning HA No HA	68 41	76.70(22.43) 73.58(22.50)	81.25 75.00		0.553	*r* = 0.06	0.580
Emotional Well-Being HA No HA	68 41	63.88(18.99) 60.80(22.03)	66.00 64.00	0.77		d = 0.15	0.442
Energy/Vitality HA No HA	68 41	46.89(21.50) 37.14(20.71)	45.00 35.00	2.33		d = 0.46	0.022*
Role Limitations Due Emotional Problems HA No HA	68 41	66.16(40.10) 64.77(35.70)	83.33 66.67		0.410	*r* = 0.04	0.682
General Health HA No HA	68 41	53.86(20.15) 46.86(21.3)	55.00 46.9	1.72		d = 0.34	0.088

Of the 67 participants that underwent puretone testing, 94% had hearing loss in at least 1 ear. Of all ears tested (*n* = 134 ears), 53% presented with sensorineural hearing loss, 37% showed mixed hearing loss, 1% showed conductive hearing loss, and 9% had hearing in the normal range (</= 20 dBHL for all test frequencies). Fifty-two participants (78%) met the criteria for hearing aid candidacy based on audiometric pure tone thresholds of 40 dB HL or greater at 500, 1000, 2000, 3000, or 4000 Hz; or thresholds 26 dB HL or greater at 3 of these frequencies [13].

Scores on the CCP and RAND SF-36 were compared between hearing aid-users and non-users that met this criterion. No significant differences were seen between subjective scores; however, both poorer- and better-ear puretone averages (PTAs) were statistically different between the groups (*p* < 0.001), with hearing aid-users having poorer thresholds and thus more severe hearing loss. The mean PTA of the poorer ear for participants that met the criteria for hearing aid candidacy and were current hearing aid users was 57.46 dB HL versus non-hearing aid users which was 40.29 dB HL. Moreover, the mean PTA of the better ear for hearing aid users was 45.65 dB HL versus 33.62 dB HL for non-users. (See Table 4).

**Table 4:** Hearing aid candidacy and CCP data

Measure	*n*	Mean(SD)	Median	t	Z	Effect Size	*P*-Value
Total CCP Score HA No HA	23 29	33.87(5.59) 34.07(5.57)	33 33	0.128		*d* = 0.04	0.899
Confidence in Ability to Hear Under Var. Cond. HA No HA	23 29	26.17(5.53) 26.69(6.15)	26 26	0.314		*d* = 0.09	0.755
Confidence in Ability to Improve Hearing Skills Using Strategies/Devices HA No HA	23 29	7.70(1.43) 7.38(1.55)	8 7		0.893	*r* = 0.12	0.37
Social Functioning HA No HA	23 29	79.35(22.17) 75.43(25.11)	87.5 75		0.374	*r* = 0.05	0.71
Emotional Well-Being HA No HA	23 29	65.04(19.31) 61.52(20.21)	68 68	0.637		*d* = 0.18	0.53
Energy/Vitality HA No HA	23 29	52.39(14.61) 44.83(22.78)	50 50		0.834	*r* = 0.12	0.404
Role Limitations Due to Emotional Problems HA No HA	23 29	65.21(40.80) 68.97(39.77)	100 66.67		0.337	*r* = 0.05	0.74
General Health HA No HA	23 29	55.65(20.36) 53.79(21.16)	60 60	0 0.320	x	*d* = 0.09	0.75

Nine individuals from the conference participants proceeded to apply for hearing aids through the Hearing Aid Project and agreed to repeat the survey protocol. All nine were fit with mid-level or premium devices by licensed audiologists in their respective areas. After at least 3 weeks of use, the 9 participants completed the online REDCap survey again. For the 9 participants fit with hearing aids, significant improvements were seen for Total CCP (*p* = 0.015) and the ‘Confidence in Ability to Hear Under Various Conditions’ subscale (*p* = 0.01). Six graduated to a higher communication confidence category. One participant demonstrated a reduction in CCP score after the intervention but reported confusion when self-administering the test online at home. A minimal clinically important difference (MCIDs) for the RAND-36 is typically in the range of 3 to 5 points, which translates into Cohen’s notion of a small effect size [17]. Given this, MCIDs were also noted for the ‘Emotional Well-Being’ and ‘Role Limitations Due to Emotional Problems’ subscales of the RAND SF-36; each showing a mean change greater than 4 points and at least a small effect size. Two-thirds of the participants fit with hearing aids increased in their overall CCP ‘Clinical Interpretation’ category (See Table 5).

**Table 5:** Pre- vs. post- measurements of 9 women tit with hearing aids

Measure	Mean Change	Z	Effect Size	*P*-Value
Total CCP Score	6.56	2.431	*r* = 0.57	0.015*
Confidence in Ability to Hear Under Various Conditions	7.33	2.558	*r* = 0.60	0.01*
Confidence in Ability to Improve Hearing Skills Using Strategies/Devices	1.00	0.552	*r* = 0.13	0.58
Social Functioning	4.17	0.276	*r* = 0.07	0.28
Emotional Well-Being	4.89	1.205	*r* = 0.28	0.23
Energy/Vitality	-7.22	0.499	*r* = 0.12	0.62
Role Limitations Due to Emotional Problems	14.81	1.000	*r* = 0.24	0.32
General Health	-1.11	0.000	*r* = 0	1.00

## Discussion

The purpose of this study was to investigate communication confidence and quality of life outcomes for individuals with Turner syndrome that have hearing loss and are treated with hearing aids versus those that are hearing aid candidates but untreated. Hearing loss in its varying types and degrees, affected the communication needs of individuals with TS in this study. There was a marked discrepancy in self-reported hearing aid candidacy and audiologist-reported candidacy for individuals at the conference. Even when recommended and given access to hearing aids, participant adoption of hearing aids remained markedly low. Addressing self-reported communication concerns using hearing aids may be one facet of improving overall communication confidence, but there is a lifelong need for healthcare providers who understand the diverse challenges and potential hearing losses associated with TS. Even within the field of audiology, understanding the changing hearing loss and needs of this population is an unmet need.

Both middle ear complications and permanent sensorineural hearing loss are highly prevalent in the TS population and remain a persistent problem throughout the lifespan. In childhood, middle ear fluid is present in 55–78% of individuals under the age of 16, with about one-third requiring pressure-equalization (PE) tubes [18]. Into puberty and early adulthood, a pattern of mid-frequency permanent sensorineural hearing loss is seen in half of individuals with TS [19, 20]. Hearing loss is progressive in nature and worsens as the individual ages. A Swedish longitudinal study found that, on average, hearing thresholds decreased 5–22 dB HL every decade, regardless of age, initial hearing status, or karyotype [21]. Overall, the research shows a clear need for continual audiological monitoring in this population. There is no equivalent research in the US, and this study is one component of that larger need.

Clinically, hearing loss can impact both speech clarity and recognition of speech sounds. Background noise, reverberation, and multiple talkers can further exacerbate a hearing loss, making communication in daily life difficult [22]. Hearing aids may improve these deficits by selectively amplifying the frequencies of difficulty and improving access to speech sounds, but settings should be set up to best support individual preference [23]. Although there is a clear need for audiological intervention in the population with TS, the number of those fit with hearing aids remains low. In a sample of 64 young Swedish adults, 52% had a confirmed hearing loss, but only 12.5% wore a hearing aid [7]. For middle-aged Swedish population with TS, a study of 44 participants showed that 91% will have a hearing loss greater than 20 dB HL; however, only 27% wear at least one hearing aid, and only 6.8% wear 2 hearing aids [24]. Low hearing aid adoption in the population with TS is not only problematic for daily functional listening, but untreated hearing loss is also correlated with several additional concerns. There is a need to better understand the barriers of accessing hearing aids so that adoption may improve globally.

Untreated hearing loss is not only a problem for communication, it is linked to higher rates of anxiety, depression, and reduced quality of life overall [25]. In adults, hearing loss is also associated with higher rates of unemployment and lower grades of employment [26]. It is important to note that in the TS population, hearing status is compounded by accompanying emotional and psychosocial problems. Individuals with TS have higher rates of lifetime depression and lower self-esteem than peers without TS [27]. The relationship between hearing loss and these emotional and psychosocial measures remains unclear; however, in a cross-sectional study, hearing impairment was shown to be statistically significantly correlated to a lower score on the Psychological General Well-Being index [28].

There remains an existing bias across global communities when it comes to individuals of low socioeconomic status and this divide is advanced further for individuals with TS experiencing emotional and psychosocial problems. This may lead to worse healthcare services and outcomes, therefore it is important for healthcare providers to understand not only foundational information on rare conditions like TS, but how their implicit bias shapes healthcare delivery to these individuals [27, 29].

Overall, the data are mixed regarding the effect of hearing aid use on communication confidence and health-related quality of life. For participants self-reporting a diagnosis of hearing loss warranting a HA recommendation, those currently wearing HAs showed significantly greater Total CCP scores. They also showed significantly greater scores for the ‘Confidence in Ability to Hear Under Various Conditions’ and ‘Energy/Vitality’ subscales.

However, for participants who underwent puretone testing at the TSSUS conference and met the hearing aid candidacy criteria described above, these differences were not seen. This may be, at least in part, explained by the fact that those currently wearing hearing aids had more severe hearing losses. Moreover, information was not available about the age or condition of each participant’s current hearing aids, hours of daily hearing aid use, nor the quality of the hearing aid fitting.

There are likely differences in the population self-reporting a known hearing loss that warranted hearing aids, and those identified at the TSSUS national convention as such. The average time to treatment adherence for hearing aids nationally is around 9 years following hearing loss identification [30]. Understanding the timeline between initial diagnosis and treatment with hearing aids might help to explain this discrepancy in future comparisons.

For the 9 participants newly fit with hearing aids, Total CCP and ‘Confidence in Ability to Hear Under Various Conditions’ subscale scores increased significantly after intervention. Additionally, changes in ‘Emotional Well-Being’, and ‘Role Limitations Due to Emotional Problems’ subscales demonstrated a minimal clinically important difference. Two-thirds of the participants fit with hearing aids increased in overall CCP category; importantly, none made it to the ‘confident’ category after treatment.

Overall results suggest that hearing aids alone are likely not sufficient at treating the communication deficits seen in the TS population. Hearing aids remain a vital component to remediating communication deficits but given their use did not bring communication confidence to its highest report, future studies may seek to evaluate the remaining challenges in confidence. Given previous data highlights a prevalence of emotional and psychosocial challenges in the TS population, these factors may negatively interact with the benefits seen in hearing aid use in this data. We present the need for interdisciplinary care and aural rehabilitation for better treatment of hearing loss and the communication deficits that accompany. Beyond the consideration of interdisciplinary care, there is a great deficit in the educational training for most healthcare providers in the United States as it relates to understanding the clinical implication of complex genetic conditions like TS. Increasing rare-disease curriculum may be an important step to improving ultimately the care of genetically-diverse individuals with complex medical needs like those seen in TS [31].

In summary of the data presented, individuals with TS in the United States are likely to benefit from amplification, but this alone will not fully remediate the low communication confidence that is expected with hearing loss. There is a further need for advancing the rare genetic disease education and a need for interdisciplinary care to best support the TS population. The data presented indicate a disparity in self-reported hearing aid candidacy and provider-determined hearing aid candidacy which highlights the importance of communicating audiological test results and recommendations in a way that promotes understanding and adherence.

### Limitations of the current study

The current survey design included self-report demographic data for web-based participation and interviewed demographic survey data for in-person participation. Self-report data was based purely on the participants’ understanding of the survey without guidance from the research team which raises a risk of question interpretation biases.

For future work in this area, hearing loss severity information should be expanded beyond conference participants. There remains a need for a national registry of data related to rare genetic conditions to better help members of the population and their providers predict healthcare patterns and improve implementation of care. Additionally, average daily use of hearing aids would be an important factor to consider as it impacts communication confidence. Separation by karyotype was not included at this stage in our investigation, but future work should address group differences and specific needs. These data support the importance of hearing loss intervention in individuals with TS, and future work may expand into specific counseling models, early versus late amplification, and functional hearing ability through decades of life. In addition to intervention by audiologists, these data support the inter-disciplinary approach to care including ear nose and throat (ENT) physicians and psychologists as important partners given the high percentages of conductive hearing loss and psychological burden of communication deficits.

## Data Availability

Data cannot be openly shared due to the need to protect study participant privacy, it can only be provided upon data use agreement in a de-identified format by contacting the corresponding author at lmann2@kumc.edu. KU Medical Center IRB contact is available at researchadministration@kumc.edu.

## Declarations

All participants signed consent forms and information shared is deidentified.

## Abbreviations

TS: Turner syndrome
